# Intraoperative indocyanine green versus clinical assessment for prevention of free flap failure: A systematic review and meta–analysis

**DOI:** 10.1016/j.jpra.2026.07.019

**Published:** 2026-07-22

**Authors:** Lawson Faulkner, Duncan Smith, Sue Liu

**Affiliations:** Warwick Medical School, University of Warwick, Coventry, CV4 7AL, UK

**Keywords:** Indocyanine green, Intraoperative, Free flap, Flap loss, Reoperation

## Abstract

**Background:**

Both total and partial flap failure are complications of free–tissue transfer surgery associated with significant morbidity, reoperation burden, and healthcare costs. This meta–analysis evaluates whether intraoperative indocyanine green fluorescence angiography (ICG–FA) differs from clinical assessment in preventing total and partial flap loss in free flap surgery.

**Methods:**

MEDLINE, Embase, Cochrane, Scopus, and Web of Science databases were searched from inception to 17th February 2026. Key search terms included free flap, free tissue transfer, indocyanine green, and fluorescence angiography. Sensitivity analysis was carried out using a leave–one–out approach. Risk of bias was assessed using ROBINS–I for non–randomised studies, and Cochrane RoB–2 for randomised trials. The analysis was pre–registered on PROSPERO (CRD420261309021).

**Results:**

Eight studies were included in the final analysis, totalling 2179 free–flap procedures, 1098 of which were controls. Controlled studies comparing intraoperative ICG–FA to clinical assessment produced 90% reduction in odds for partial failure (OR 0.10, 95% CI: 0.04, 0.26; p < 0.01), 41% reduction for total failure (OR 0.59, 95% CI: 0.35, 0.97; p = 0.04), and 53% reduction for requirement of reoperation (OR 0.47, 95% CI: 0.25, 0.88; p = 0.02).

**Conclusion:**

Intraoperative ICG–FA was associated with reduced odds of partial and total flap loss, as well as reoperation, compared with conventional clinical assessment. These findings suggest that ICG–FA may therefore represent a valuable adjunct in free flap surgery. Further high–quality prospective trials will be required to better elucidate the true magnitude of effect of ICG–FA in free flap surgery and clarify its role in contemporary microsurgical practice.

## Introduction

Free–tissue transfer has become a cornerstone of modern reconstructive surgery; however, with it comes the potential for flap failure. Flap failure remains a rare but catastrophic complication with reported incidence varying from according to anatomical site, patient comorbidity, and institutional expertise^1^, ^2^, ^3^ Flap loss is associated with significant morbidity, prolonged hospital stays, increased healthcare costs, and the potential need for further complex reconstruction.^4^ Several factors can be responsible aetiological factors, however vascular compromise has been implicated as by far the most significant,^1^^,^^5^ reflecting the critical importance of uninterrupted arterial inflow and venous outflow during the early postoperative period until neovascularisation occurs.^6^

Current practice for the intraoperative assessment of free flaps varies between centres and surgeons. The traditional evaluation relies primarily on clinical assessment, which assesses flap colour, temperature, capillary refill, and bleeding at the wound edge.^7^ Whilst readily available and low–cost, these measures rely on clinical judgement and may not reliably detect early microvascular perfusion deficits prior to overt vascular compromise.^8^ In response to this, a range of adjunctive technologies have been explored to provide a more objective assessment of flap perfusion intraoperatively,^9^^,^^10^ of which indocyanine green fluorescence angiography (ICG–FA) has seen increasing attention in recent years.^11^

ICG is a tricarbocyanine compound administered intraoperatively as an intravenous bolus. It readily binds plasma proteins within the intravascular compartment, enabling real–time visualisation of tissue perfusion through fluorescing under near–infrared light.^12^^,^^13^ It is rapidly taken up and excreted in the bile by the liver with a half–life of approximately 3–4 min,^12^^,^^13^ with an excellent safety profile and a very low incidence of adverse events.^12^^,^^14^ ICG has therefore been explored for various surgical applications,^13^^,^^15^ but in the context of free tissue transfer, has been used to aid identification of perforators; after flap elevation to identify non–viable flaps or trim avascular portions of flaps; and post–operatively to monitor flap performance.^16^

Despite increasing surgical integration of ICG–FA in reconstructive surgery, there is a paucity of any focussed meta–analyses examining its role in the intraoperative assessment of free flaps. Therefore, the focus of the present review is on these intraoperative applications of ICG–FA and its potential impact on both partial and total flap failure.

## Methods

### Search strategy

The literature search utilising MEDLINE, Embase, Cochrane, Scopus, and Web of Science was carried out from inception to 05th December 2025. Additional searches were carried out on 17th February 2026 to ensure all recent relevant papers had been included. Additional systematic reviews were also screened to ensure comprehensive identification of eligible studies^9^^,^^17^, ^18^, ^19^ The following Boolean operators were utilised in the search: (Free Tissue Flap OR Free Flap OR Free Tissue OR Free Tissue Transfer) AND (Indocyanine Green OR Fluorescence Angiography) with truncation and explosion of terms as appropriate to maximise retrieval. The analysis was registered on PROSPERO (CRD420261309021).

### Study selection

Studies were screened independently by two reviewers, LF & DS, with a third reviewer deferred to if needed to resolve disagreements. Included articles were those that utilised ICG–FA to assess intraoperative perfusion of free flaps for correction of soft–tissue or bony defects, whether due to congenital or acquired deformity. Excluded studies included those without controls, utilising multiple intraoperative modalities for perfusion assessment, if intraoperative assessment focussed on other parameters such as lymphatic flow following lymphoedema correction instead of flap failure, if they did not report flap failure rate, or if they involved children.

### Data extraction and risk of bias assessment

Primary outcome measures were the occurrence of total or partial flap loss, as well as requirement for reoperation, in flaps assessed intraoperatively by ICG–FA. Data were independently extracted by two reviewers, LF & DS, in accordance with the pre–defined study–selection criteria and collated in a standardised Excel sheet (see supplementary information). Data extracted included the study design, numbers of participants and controls, demographic data, imaging system used, any reported financial costs, flap failures, indications for surgery, flap types, and defect locations.

Risk of Bias assessment was carried out in duplicate by two reviewers (LF & SL) using Risk of Bias in Non–randomized Studies or Interventions (ROBINS–I)^20^ for controlled studies, and Cochrane Risk of Bias 2 (RoB–2)^21^ for randomised controlled trials (RCTs).

### Statistical analysis

Statistical analysis was carried out using STATA 18.0 (StataCorp, College Station, TX, USA). The difference in flap failure and need for reoperation was compared between patients who were assessed intraoperatively with indocyanine green and those who were assessed using traditional clinical assessment. Binary outcomes were assessed using odds ratio (OR) with associated 95% confidence intervals (CIs). Pooled effect estimates were calculated using a random–effects meta analysis with restricted maximum likelihood (REML) to account for between–study heterogeneity arising from differences in patient demographics, surgical technique, and procedural characteristics between studies. Inter–study variance was estimated and reported as τ². Statistical heterogeneity was assessed using inconsistency statistic (I^2^). Overall pooled effect sizes were evaluated using a z–test, with statistical significance set at p < 0.05. Sensitivity analyses were carried out with a leave–one–out approach to assess the robustness of the pooled effect.

## Results

### Study selection

**Fig. 1:**
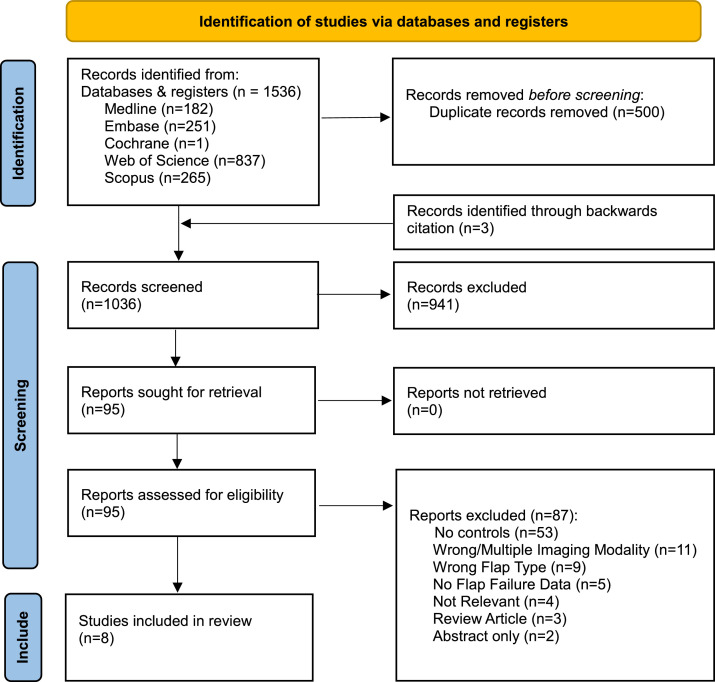
PRISMA 2020 flow chart showing inclusion of studies for meta–analysis.

A total of 1536 records were identified, which after initial deduplication left 1036. Following title and abstract screening 95 were left for full text screening which once completed left eight papers^22^, ^23^, ^24^, ^25^, ^26^, ^27^, ^28^, ^29^ for the final analysis. Backwards citation identified three additional papers for possible inclusion, however none contained control groups for comparison (Fig. 1).

All articles were single–centre studies and spanned four nations. There were additional case–controlled studies which had to be excluded due to unclear reporting of flap failure rates,^30^ or not separating out free–flap data to those from pedicled flaps.^7^^,^^31^^,^^32^

Details of Intraoperative ICG Technologies

Whilst many ICG–FA technologies are available commerically, four were used by the seven included studies, all of which are FDA approved (Table 1).

**Table 1:** Summary of the perfusion technologies currently in use with their relative advantages and disadvantages. Equipment used was extracted from all studies included in the analysis. *Quest values are based on the FDA approved device, but offer bespoke customisation options for devices at point of sale.

ICG Technology	Device Used By	Device Configuration	Resolution (pixels)	Maximum Frame Rate (FPS)	Field of View (cm^2^)	Colour	Working Distance	Excitation Wavelength
SPY Elite; Novadaq Spy, Stryker©, Kalamazoo, Michigan, United States.	Choudhary 202,3^23^, Green 201,3^25^, Momeni 202,0^27^, Yoo 202,2^28^	Overhead Arm–Mounted	1024×768	30	18.5 × 13.5	Greyscale	30cm	805nm
Fluobeam, Fluoptics, Grenoble, France	Didzun 202,5^24^	Handheld	720×576	25	2.2 × 1.5 to 20×14	Greyscale	15–25cm	680–750nm
Quest Spectrum*, Quest Medical Imaging©, Wieringerwerf, The Netherlands	Michi 202,2^26^	Overhead Arm–Mounted or Handheld	Not Specified	20	2.2 × 2.2	Full–Colour & Greyscale	30cm	700–1000nm
Photo Dynamic Eye, Hamamatsu, Shizuoka, Japan	Varela 2020^29^	Handheld	640×480	20	5 × 5 to 10×6.7	Greyscale	30cm	760nm

### Operative outcomes & demographics

The primary outcome measure of flap total failure was 2.4% overall for the use of ICG and 3.9% for flaps assessed with clinical assessment alone. Partial failure rates were 0.9% and 8.5% overall for ICG and No–ICG groups, respectively. Average BMI was similar for both ICG (29.7) and No–ICG (29.5) groups, whilst average patient ages were 54.6 for cases and 53.7 for controls (Table 2).

**Table 2:** Patient characteristics of all studies included. BMI, Body Mass Index. NR = Not reported.

Study	Free Flaps n		Total Failure n(%)		Partial Failure n(%)		Male:Female n:n		Mean Age years		Mean BMI kg/m^2^	
	ICG	No–ICG	ICG	No–ICG	ICG	No–ICG	ICG	No–ICG	ICG	No–ICG	ICG	No–ICG
Chen 2023	259	261	10 (3.8)	22 (8.4)	NR	NR	245:14	240:21	57	56	NR	NR
Choudhary 2023	438	439	6 (1.4)	9 (2.0)	2 (0.5)	22 (5.0)	346:92	356:83	53	50	NR	NR
Didzun 2025	36	71	0 (0)	1 (1.4)	1 (2.8)	13 (18.3)	26:10	42:19	49	61	28	30
Green 2013	55	29	1 (1.8)	1 (3.4)	1 (1.8)	3 (10.3)	NR	NR	NR	NR	NR	NR
Michi 2022	24	24	1 (4.1)	0 (0)	1 (4.2)	8 (33.3)	0:24	0:24	52	54	26	26
Momeni 2020	58	79	0 (0)	1 (1.3)	NR	NR	0:58	0:79	52	50	28	29
Varela 2020	24	27	0 (0.0)	0 (0.0)	0 (0.0)	4 (14.8)	0:24	0:27	52	48	25.4	24.7
Yoo 2022	187	168	7 (3.7)	8 (4.7)	NR	NR	0:187	0:168	58	59	31	30
**OVERALL**	1081	1098	25 (2.4)	42 (3.9)	5 (0.9)	50 (8.5)	617:409	638:421	54.6	53.7	29.7	29.5

Studies included 565 DIEPs, 338 Radial Forearm Flaps, 580 Anterolateral thigh flaps, 342 fibula free flaps, 134 latissimus dorsi ± parascapular free flaps, 89 msTRAM, 19 medial sural artery flaps, and 112 classified as ‘other’. Recipient regions included 1229 in the head and neck, 657 breast, 55 upper limb, 147 lower limb, 18 trunk, and 73 unspecified. 1844 flaps were carried out for oncologic purposes, 141 due to trauma, 20 due to infection, and 174 were unclassified (Table 3).

**Table 3:** Operative characteristics of all studies included in the analysis. ALT, anteiror lateral thigh; RFF, radial forearm flap; FFF, Fibular Free Flap; MSA, medial sural artery; LDF, Latissimus Dorsi Free Flap; DIEP, Deep Inferior Epigastric Perforator; msTRAM, Muscle–Sparing Transverse Rectus Abdominis Myocutaneous; PAP, Profunda Artery Perforato.

Author	Study Type	Country	Flap Types	Recipient Sites	Reason for Surgery	Alternative Measuring Techniques (No–ICG Group)
Chen 2023	Retrospective Cohort, Single–centre	Taiwan	ALT, RFF, FFF, MSA	Head & Neck	Malignancy	Clinical assessment, a pinprick test, and a handheld Doppler device.
Choudhary 2023	Retrospective Cohort, Single–centre	India	ALT, RFF, FFF, DIEP	Head & Neck, Breast, Upper Limb, Lower Limb, Trunk	Malignancy, Trauma	NR
Didzun 2025	Prospective Cohort, Single–centre	Germany	LDFF	Head & Neck, Upper Limb, Lower Limb, Trunk	Malignancy, Trauma, Infection, Chronic Wound	Clinical evaluation comprised observations of capillary blanching and refill, Skin Color, and Bleeding.
Green 2013	Retrospective Cohort, Single–centre	USA	ALT, FFF, LDFF, Gracilis, Serratus, Lateral Arm	Head & Neck, Upper Limb, Lower Limb	NR	NR
Michi 2022	Prospective Cohort with Retrospective controls, Single–centre	Netherlands	DIEP	Breast	Malignancy	NR
Momeni 2020	Prospective Cohort, Single–centre	USA	DIEP, MS–TRAM, PAP	Breast	Malignancy	NR
Varela 2020	Randomised Controlled Trial, Single–Centre	Spain	DIEP	Breast	Malignancy	Clinical criterion for tissue excision was based on blue coloration with rapid capillary refilling.
Yoo 2022	Retrospective Cohort, Single–centre	USA	DIEP	Breast	Malignancy	Flap color, capillary refill, Doppler Signals, and Punctate Bleeding.

### Meta–analysis

**Fig. 2:**
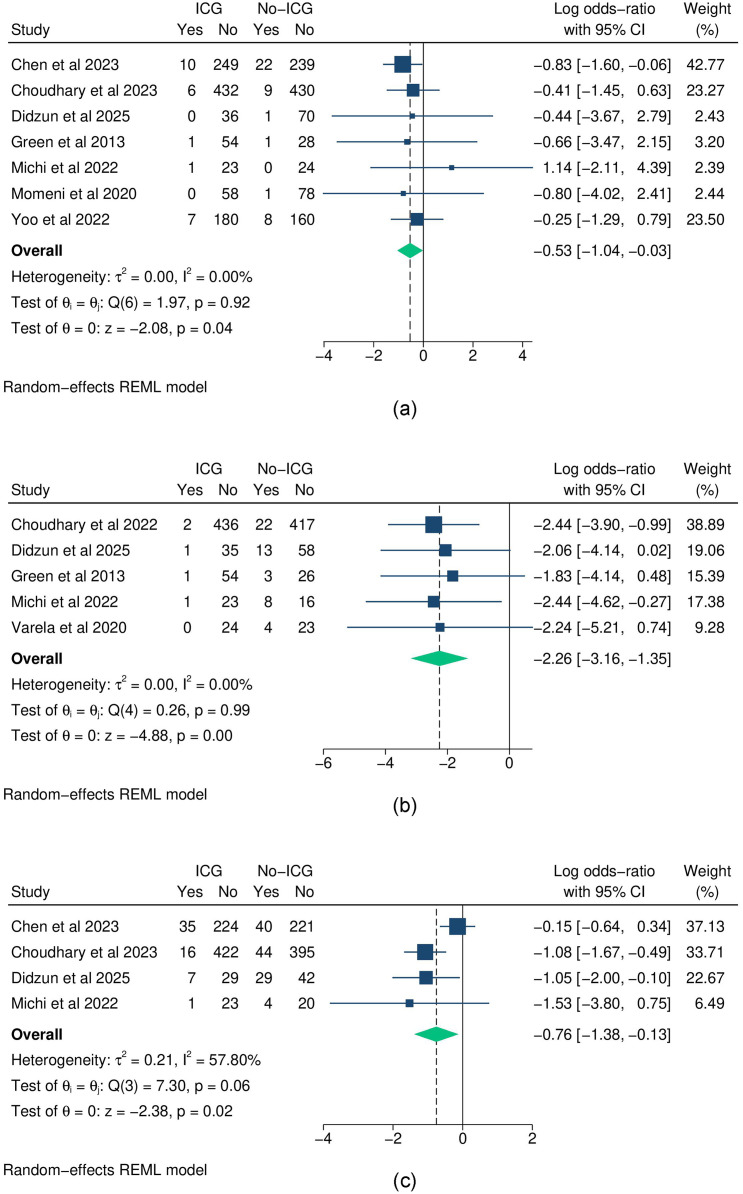
Forest plot for (A) total flap loss, (B) partial flap loss, (C) requiring repoperation in case–controlled studies comparing ICG to cinical assessment.

Meta analysis outcomes are shown as forest plots in Fig. 2. Pooled odds ratios (OR) for total flap failure with the use of ICG–FA compared to clinical assessment was 0.59 (95% CI: 0.35, 0.97; p = 0.04) with no significant inter–study variance (T^2^=0.00) or heterogeneity (I^2^=0.00) identified. OR for partial flap failure was 0.10 (95% CI: 0.04, 0.26; p = 0.00) with no significant inter–study variance (T^2^=0.00) or heterogeneity (I^2^=0.00) identified. OR for requirement of reoperation was 0.47 (95% CI: 0.25, 0.88; p = 0.02), with significant interstudy variance (T^2^=0.21) and greater heterogeneity identified (I^2^=57.80%).

### Sensitivity analysis

**Fig. 3:**
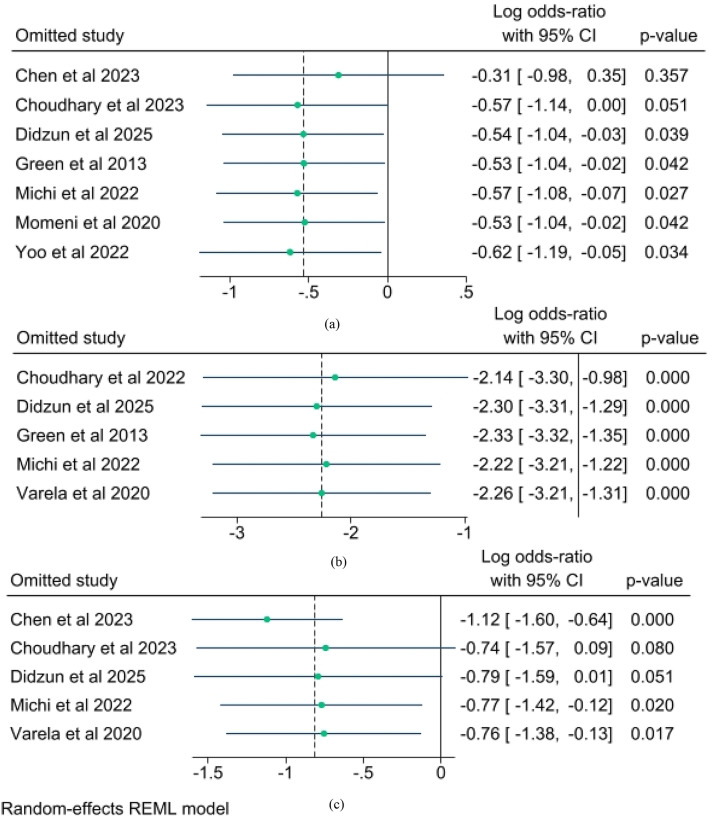
Leave–one–out analysis for (A) total flap loss, (B) partial flap loss, (C) requiring reoperation.

A Leave–One–Out meta–analysis was conducted for total loss, partial loss, and requirement of reoperation (Fig. 3). For partial flap failure, the direction and magnitude of pooled estimated effect did not change upon removal of any of the studies. For total flap failure, the overall estimated effect remained in the same direction after removal of all the studies; however, removal of both Chen et al. and Choudhary et al. did affect the significance of the outcome. Similarly for requirement of reoperation, the overall direction of pooled effect remained the same following removal of all studies, but significance was affected after removal of Choudhary et al. and Didzun et al.

### Risk of bias

**Fig. 4:**
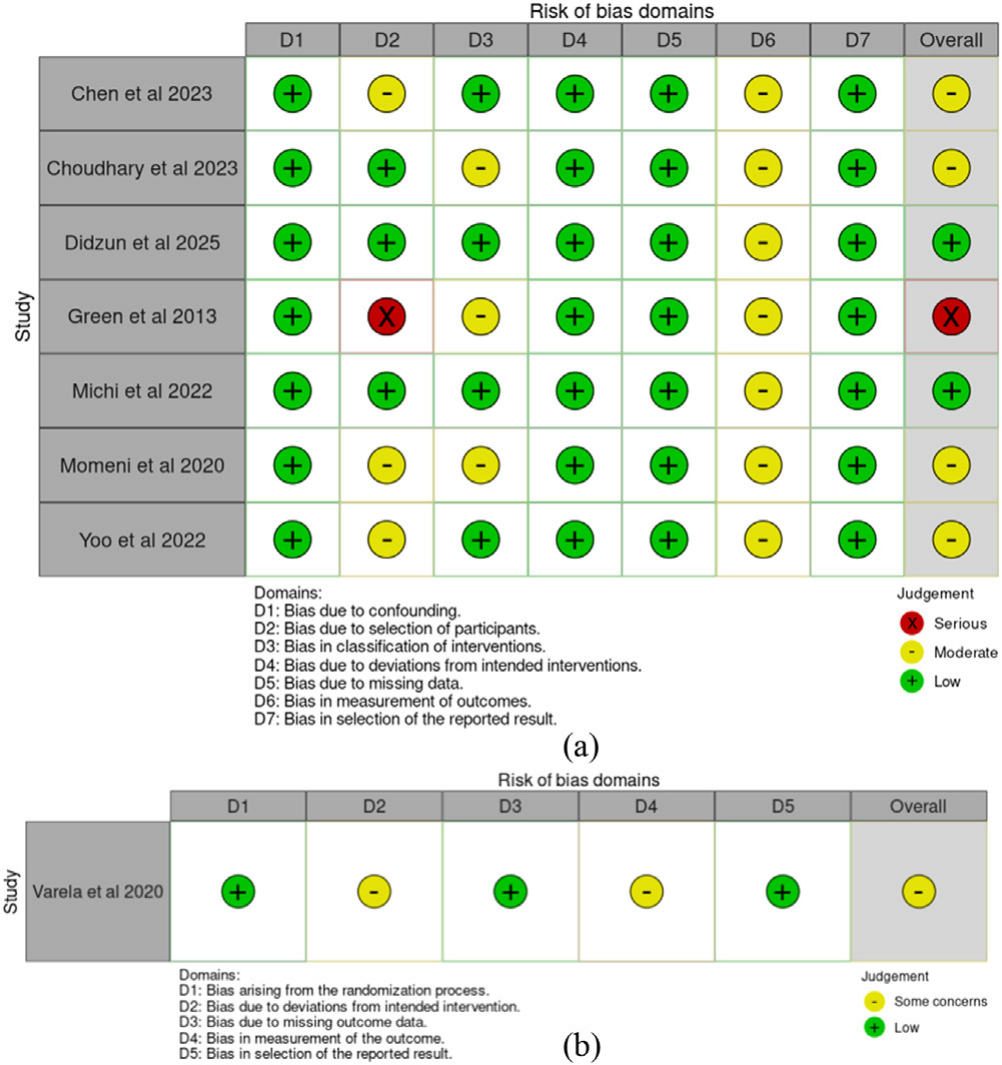
Risk of bias summary plot for ROBINS–1 assessment of Non–randomised controlled trials (A) and ROB–2 for randomised controlled trials (B).

The Risk of Bias In Non–Randomised Studies (ROBINS–I) and Cochrane Risk of Bias 2 (ROB–2) tools were used for assessment of risk of bias (Fig. 4). ROBINS–I is a validated and widely used tool for evaluating risk of bias in non–randomised studies of interventions, which assesses publication bias across seven methodological domains.^20^ Two studies were found to have low risk of bias, four studies moderate risk, and one serious risk. ROB–2 is a similar tool for assessing Randomised Controlled Trials (RCTs) which assess studies across five methodological domains. For the single included RCT, the only potential issue identified was that surgeons were aware of the intervention on the morning of the surgery; however, because there were no discernable deviations that arose from this knowledge, missing outcomes, measurement of otucomes, or selection of the reported result, it was still considered low risk.

## Discussion

To our knowledge, this review presents the first meta–analysis evaluating intraoperative ICG–FA for the prevention of free–flap failure across multiple reconstructive contexts. Pooled analysis across the included studies demonstrated that ICG–FA significantly reduced the odds of both partial and total flap failure, as well as the need for reoperation, in comparison to standard intraoperative assessment.

Previous literature has largely supported these findings, albeit without sufficient evidence to draw definitive conclusions regarding flap failure. A 2022 Delphi survey^11^ spanning four continents and involving 22 international experts reached absolute consensus that ICG should be available for all plastic and reconstructive surgery services, highlighting strong professional support for the technology despite the still–maturing evidence base. Prior reviews on the subject have similarly reported favourable outcomes associated with ICG–FA use; however, these were primarily limited by a paucity of comparative data and an inability to quantitatively assess its effect on flap survival. Parmeshwar et al.^33^ evaluated 352 free flaps compared with non–ICG controls and suggested that ICG may be more sensitive than clinical assessment when assessing flap perfusion, but lacked substantial evidence at the time to draw a significant conclusion about its influence on total or partial flap failure. Notably, one of the three studies included in their flap–failure analysis, Duggal et al.^7^ did not differentiate between free and pedicled flaps when reporting total and partial flap failures. Consequently, in accordance with our predefined inclusion criteria, it was not eligible for inclusion in the present meta–analysis.

Earlier, Liu et al.^18^ provided a comprehensive narrative synthesis of 17 case–series examining the broader role of ICG in reconstructive surgery, highlighting its potential for aiding intraoperative decision making; however, no quantitative synthesis was performed, and no conclusions were drawn regarding its impact on flap failure outcomes. Since this review was published, several groups have published larger case series which appear to support the rates seen in the two–armed studies of this review. Dornseifer et al.^34^ retrospectively evaluated 126 ICG–monitored free flaps reporting total flap failure of 4%, while Steiner et al.^35^ reported lower occurrence of 2.3% in 217 flaps, and Ludolph et al.^36^ even lower with zero total or partial failures across 100 free flaps.

More recently, Panettella et al.^37^ published a larger series of 400 flaps, 257 of which were free flaps, reporting no cases of total flap failure, and two cases of partial failure (0.8%). The group demonstrated the importance of utilising ICG–FA at several stages throughout the procedure, as well as the key utility of ICG–FA in exposing mal–perfused regions of the flap for possible resection. Out of the many implementations of ICG intraoperatively, it may be this particular ability to identify the outer boundary of perforator blood supply that could potentially explain the dramatic effects seen on incidence of partial flap failure observed in the present meta–analysis. This may also explain why ICG–FA sees a less dramatic effect on total flap failure. Whilst it can flag immediately compromised flaps, it can do little to predict acute postoperative events, such as thrombosis or arterial occlusion, that often lead to a total failure, which are also strongly influenced by patient–related factors, such as obesity, smoking status, and peripheral arterial disease,^5^ rather than intraoperatively–determined flap margins.

Panettella et al. also explored the utility of ICG–FA in predicting venous congestion of flaps. They reported that a flap may either glow brighter than surrounding tissues, due to buildup of ICG within flap, or not at all, in the case contrast is administered later and the flap has already become congested with contrast–free blood. Indeed, this latter variation was also reported by Yoshimatsu et al.^38^^,^^39^ who found a lack of fluorescence of the flap to indicate venous congestion – which could be differentiated from arterial insufficiency by bleeding following pinprick testing. It is worth noting, however, that the typical washout period is around 20–30 min,^12^^,^^40^ which could render repeated measurements to assess for venous insufficiency cumbersome in everyday surgical practice. Nevertheless, as standard ICG procedure already involves assessments at several operative timepoints, which are inherently separated by sufficient time to allow dye clearance, it may be that minor modifications to operative workflow could permit more structured integration of ICG–FA at additional stages of the procedure. Further studies optimising dosing strategies and clearance intervals,^41^ may also enhance feasibility and its use for dynamic intraoperative assessment of venous outflow. Moreover, further research is required to better characterise the fluorescence patterns associated with venous insufficiency and to establish reproducible diagnostic criteria for its intraoperative identification.

An important consideration for the implementation of any technology is that of cost. Several analyses have attempted to evaluate the cost–effectiveness of introducing ICG into microsurgical practice, although most have focussed on free–flap breast surgery thus far. Chatterjee et al.^42^ found that ICG was more cost–effective than clinical assessment when the absolute incidence of complications was 4% or more, with complications defined as total or partial flap loss, mastectomy flap necrosis, seroma, infection, or hematoma. Duggal et al.^7^ similarly reported cost–savings associated with routine use of ICG, without identifying specific criteria to guide patient selection. In contrast, Kanuri et al.^43^ found that indiscriminate use of ICG did not confer cost–savings; however, selective use in higher–risk groups such as those with a body mass index >30, smokers, or those with mastectomy weights over 800 g, was associated with cost savings. Given the paucity of recent cost–analyses of ICG in free–flap surgery, particularly outside the context of breast reconstruction, it is clear that further contemporary cost–benefit analyses are required.

The primary limitation of this meta–analysis is the relatively limited number of studies, several of which are further constrained by somewhat modest sample sizes. Although each outcome analysis reached statistical significance, limited numbers reduced statistical power, and pooled estimates may therefore be influenced by individual study effects. In addition, although a random–effects model was employed, the predominance of non–randomised controlled studies means that heterogeneity in study design, operative technique, timing of ICG administration, and outcome definitions may have contributed to variability across analyses. We were also unable to meaningfully compare specific mechanisms of flap failure as these outcomes were inconsistently reported and lacked standardised definitions. Inconsistent reporting and separation of outcomes by flap type, anatomical location, and surgical indication also prevented more granular subgroup analyses. These variables are important factors for consideration as flap types and locations are associated with varying baseline risks of flap loss.^1^ Furthermore, the inherent size of different flap types included within the studies would likely confer different risks of flap failure, particularly when considering partial flap failure. Future research should prioritise transparent and standardised reporting of flap–related complications, including stratified outcomes by flap type, anatomical location, and surgical indication, to enable more granular pooled and subgroup analyses and strengthen the accumulating evidence base in this field.

In addition to the limitations inherent to this meta–analysis, the existing literature remains predominantly observational in nature, with a paucity of high–quality prospective or randomised comparative studies analysing ICG or comparing it to other adjunctive interventions. Our analysis was therefore limited to comparisons between ICG–FA and clinical assessment, and did not evaluate its relative performance against other adjunctive perfusion assessment technologies. Further to this, there remains no standardised framework for reporting of flap perfusion in studies evaluating ICG–FA. Considerable variation exists within the literature in how fluorescence intensity is quantified, whether absolute or relative perfusion thresholds are applied, timing of image acquisition following administration of ICG, and how perfusion deficits are defined. This lacking consensus limits inter–study comparability and hampers analysis of clinically meaningful reference values that could be correlated with viability and risk of complication. Direct comparisons between intraoperative monitoring modalities, cost–effectiveness analyses, and the establishment of standardised perfusion thresholds will be essential to define the optimal role of ICG–FA within contemporary microsurgical practice.

In conclusion, the present meta–analysis suggests that ICG could be a valuable adjunct to conventional clinical assessment in free flap surgery, having produced significant reductions in the rates of partial and total flap failure, as well as incidence of reoperation. Its most pronounced benefit appears to lie in identifying poorly perfused peripheral tissue, thereby helping to prevent partial necrosis following free–tissue transfer. Further prospective studies are needed to standardise protocols and optimise integration into operative workflow, but current evidence supports its role in improving free flap outcomes.

## Author contributions

Conception and design: LF, Literature search, study selection, and data extraction LF & DS, Methodological appraisal: LF, DS, SL. Statistical analysis: LF, DS, SL. Writing and Critical revision of the article: LF, DS, SL.

## Declaration of AI and AI–assisted technologies in the writing process

During the preparation of this work the authors used ChatGPT in order to suggest refinements of some parts of the text to improve clarity and readability. After using this tool, the authors reviewed and edited the content as needed and take full responsibility for the content of the publication.

## Funding information

No sources of funding to declare.

## Conflicts of Interest

No conflicts of interest to declare.

## Data Availability

Data used for the present meta–analysis can be found in the supplementary information (supplementary Tables 2a and 2b).

## Appendix Supplementary materials

Image, application 1
