# Supplementary material for: Intraoperative indocyanine green versus clinical assessment for prevention of free flap failure: A systematic review and meta–analysis
Source: JPRAS Open. 2026 Jul 22;51:616–29. doi: 10.1016/j.jpra.2026.07.019 (PMC13486775; doi:10.1016/j.jpra.2026.07.019)
Supplement: Supplementary file 1 [file mmc1.docx]

***Supplementary Tables 2a & 2b detailing the dataset used for the review article:***

| **Author** | **Year** | **Country** | **Study Design** | **No. Study Centres** | **Total Flaps** | **Total Patients** | **Cases ICG Flaps** | **Controls No-ICG Flaps** | **Case Male** | **Case Female** | **Cont male** | **Cont Female** | **AVG Age Cases** | **AVG Age Controls** | **AVG BMI Case** | **AVG BMI Control** | **Imaging System** | **Total Flap Loss Cases** | **Partial Flap Loss Cases** | **Failure Control** | **Partial Failure Control** | **Rexploration/Reoperation Cases** | **Rexploration/Reoperation Control** |
| --- | --- | --- | --- | --- | --- | --- | --- | --- | --- | --- | --- | --- | --- | --- | --- | --- | --- | --- | --- | --- | --- | --- | --- |
| Chen et al 2023 | 2023 | Taiwan | Retrospective Cohort | Single | 520 | 486 | 259 | 261 | 245 | 14 | 240 | 21 | 57.08 | 56.39 | N/A | N/A | N/A | 10 | N/A | 22 | N/A | 35 | 40 |
| Choudhary et al 2023 | 2023 | India | Retrospective Case Series with Comparators | Single | 877 | 875 | 438 | 439 | 346 | 92 | 356 | 83 | 53 | 50 | NA | NA | SPY Elite system | 6 | 2 | 9 | 22 | 16 | 44 |
| Didzun et al 2025 | 2025 | Germany | Prospective Cohort | Single | 107 | 107 | 36 | 71 | 26 | 10 | 42 | 19 | 49 | 61.3 | 27.9 | 29.6 | Fluoptics Fluobeam; Fluoptics, France | 0 | 1 | 1 | 13 | 7 | 29 |
| Green et al 2013 | 2013 | USA | Prospective Study with Retrospective Controls | Single | 84 | 84 | 55 | 29 | NA | NA | NA | NA | NA | NA | NA | NA | Lifecell SPY Elite imaging, Lifecell Corporation, Bridgewater, NJ) | 1 | 1 | 1 | 3 | N/A | N/A |
| Michi et al 2022 | 2022 | Netherlands | Prospective Cohort with Retrospective Controls | Single | 48 | 48 | 24 | 24 | N/A | 24 | N/A | 24 | 52.1 | 54.4 | 26.2 | 26.4 | NIRF imaging system (Quest Medical Imaging, Middenmeer, the Netherlands) | 1 | 1 | 0 | 8 | 1 | 4 |
| Momeni et al 2020 | 2020 | USA | Retrospective observational cohort study | Single | 137 | 80 | 58 | 79 | N/A | N/A | N/A | N/A | 52 | 49.9 | 28.3 | 29.5 | SPY Elite | 0 | N/A | 1 | N/A | N/A | N/A |
| Varela et al 2020 | 2020 | Spain | Randomised Controlled Trial | Single | 51 | 51 | 24 | 27 | 0 | 24 | 0 | 27 | 52 | 48 | 25.4 | 24.7 | Photo Dynamic Eye (Pulsion Medi- cal Systems) | N/A | N/A | 0 | 4 | 0 | 4 |
| Yoo et al 2022 | 2022 | USA | Retrospective Observational Study | Single | 355 | 224 | 187 | 168 | 0 | N/A | 0 | N/A | 58.4 | 58.83 | 30.89 | 30.47 | ICGA (SPY Imaging System, NOVADAQ Technologies; Mississauga, Canada) | 7 | N/A | 8 | N/A | N/A | N/A |

***Supplementary Table 2a: Dataset for the Analysis***

| **Author** | **Head & Neck** | **Breast** | **Upper Limb** | **Lower Limb** | **Trunk** | **Unspecified** | **total** | **Overall Defect Location** | **DIEP** | **Radial Forearm** | **Anterolateral Thigh** | **Medial Sural Artery** | **Fibula** | **Latissimus Dorsi +/- Parascapular** | **Others** | **Oncologic** | **Trauma** | **Infection** | **Burn** | **Other / unspecified** | **Total** |
| --- | --- | --- | --- | --- | --- | --- | --- | --- | --- | --- | --- | --- | --- | --- | --- | --- | --- | --- | --- | --- | --- |
| Chen et al 2023 | 520 | 0 | 0 | 0 | 0 | 0 | 520 | Head & Neck | 0 | 80 | 285 | 19 | 133 | N/A | 3 | 520 | 0 | 0 | 0 | 0 | 520 |
| Choudhary et al 2023 | 684.08 | 65.76 | 52.62 | 61.38 | 13.16 | 0 | 877 | Mixed | 65.76 | 258.72 | 280.67 | N/A | 206.05 | N/A | 65.8 | 706 | 87.7 | 0 | 0 | 83.3 | 877 |
| Didzun et al 2025 | 14 | 0 | 2 | 86 | 5 | 0 | 107 | Mixed | 0 | 0 | 0 | 0 | 0 | 107 | 0 | 27 | 53 | 20 | 0 | 7 | 107 |
| Green et al 2013 | 11 | NA | NA | NA | NA | 73 | 84 | Mixed | 0 | 0 | 15 | 0 | 3 | 27 | 3 gracillis, 2 vastus lateralis, 6 lateral arm, 2 serratus chimeric, 26 other | NA | NA | NA | NA | 84 | 84 |
| Michi et al 2022 | 0 | 48 | 0 | 0 | 0 | 0 | 48 | Breast | 48 | 0 | 0 | 0 | 0 | 0 | 0 | 48 | 0 | 0 | 0 | 0 | 48 |
| Momeni et al 2020 | 0 | 137 | 0 | 0 | 0 | 0 | 137 | Breast | 45 | 0 | 0 | 0 | 0 | 0 | 93 (89 msTRAM, 4 Profunda artery perforator) | 137 | 0 | 0 | 0 | 0 | 137 |
| Varela et al 2020 | 0 | 51 | 0 | 0 | 0 | 0 | 51 | Breast | 51 | 0 | 0 | 0 | 0 | 0 | 0 | 51 | 0 | 0 | 0 | 0 | 51 |
| Yoo et al 2022 | 0 | 355 | 0 | 0 | 0 | 0 | 355 | Breast | 355 | 0 | 0 | 0 | 0 | 0 | 0 | 355 | 0 | 0 | 0 | 0 | 355 |

***Supplementary Table 2b: Continued Dataset for the Analysis***
